# Perceived self-efficacy to teach comprehensive abortion care among nursing and midwifery faculty in higher learning institutions in Rwanda: A mixed method study

**DOI:** 10.1371/journal.pone.0300542

**Published:** 2024-03-18

**Authors:** Aimable Nkurunziza, Michael Habtu, Madeleine Mukeshimana, Tamrat Endale, Yvonne Delphine Nsaba Uwera, Reverien Rutayisire, Justine Bagirisano, Jean Bosco Henri Hitayezu, Marie Laetitia Bazakare Ishimwe, Jean De Dieu Uwimana

**Affiliations:** 1 Faculty of Health Sciences, Arthur Labatt Family School of Nursing, London, Canada; 2 School of Nursing and Midwifery, College of Medicine and Health Sciences, University of Rwanda, Kigali, Rwanda; 3 School of Public Health, College of Medicine and Health Sciences, University of Rwanda, Kigali, Rwanda; 4 The Center for International Reproductive Health Training at the University of Michigan (CIRHT-UM), Ann Arbor, Michigan, United States of America; 5 School of Health Sciences, College of Medicine and Health Sciences, University of Rwanda, Kigali, Rwanda; 6 The Center for International Reproductive Health Training at the University of Rwanda, Kigali, Rwanda; 7 Center for Teaching and Learning Enhancement(CTLE), University of Rwanda, Kigali, Rwanda; Western University, BOTSWANA

## Abstract

**Introduction:**

Comprehensive abortion care is an emerging intervention being integrated into nursing and midwifery curricula. Yet, no studies have been conducted in Rwanda to determine whether faculty perceive themselves as capable of teaching comprehensive abortion care. This study aims to evaluate the perceived self-efficacy to teach comprehensive abortion care among nursing and midwifery faculty in higher learning institutions in Rwanda.

**Materials and methods:**

The University of Rwanda College of Medicine and Health Sciences Institutional Review Board approved this study (UR-CMHS-IRB No 335/CMHSIRB/2022). In quantitative, a self-administered questionnaire was administered to 98 study participants. Data were entered into Statistical Package for the Social Sciences (SPSS) version 26 and analyzed using Chi-square test with a p-value of 0.05 set as the significance level. In the qualitative part, an interview guide was developed based on quantitative data to understand comprehensive abortion care teaching fully. Data were collected from four focus group discussions with eight participants in each group, entered in Dedoose, and analyzed thematically.

**Results:**

Among the 98 study participants who were invited to participate in this study, only 85 filled out the questionnaires. This translates into 86.7% of the response rate. More than half 58.8% had adequate self-efficacy in teaching comprehensive abortion care. A Chi-square test has revealed that being a male, being a midwife, and having more years of working experience in nursing education were significantly associated with self-efficacy in teaching comprehensive abortion care (p value <0.05). In the qualitative phase, 32 study participants participated in four focus group discussions and four themes were identified: a) variability in confidence levels to teach comprehensive abortion care; b) readiness about teaching comprehensive abortion care; c) facilitators of teaching comprehensive abortion care; and d) contextual challenges to teach comprehensive abortion care.

**Conclusions:**

The findings revealed that faculty’s self-efficacy in teaching comprehensive abortion care was not adequate. Personal and religious beliefs and institutional barriers were also reported to hinder self-efficacy in teaching comprehensive abortion care. Therefore, intensive comprehensive abortion care training for nursing and midwifery faculty in higher learning institutions should be provided, including values clarification and attitude transformation training for attitudes and beliefs. It is also critical for higher learning institutions to develop strategies for overcoming the challenges faculty face when teaching comprehensive abortion care.

## Introduction

Providing women and girls with comprehensive abortion care (CAC) is fundamental to achieving the Sustainable Development Goals (SDGs) related to good health and well-being (SDG3), as well as gender equality (SDG5) [1]. The lifetime risk of pregnancy-related death is higher for women in less developed countries, which have, on average, many more pregnancies. There is a 1 in 5400 chance that a 15-year-old woman will die from maternal causes during her lifetime in a high-income country, compared to a 1 in 45 chance in a low-income country [2]. There is no doubt that abortion plays a significant role in maternal mortality worldwide. Maternal deaths from unsafe abortions range from 4.7% to 13.2% yearly [3].

The number of women who die in unsafe abortions is estimated at 30 per 100 000 in developed regions and the number of deaths per 100 000 unsafe abortions increases to 220 in developing regions [4]. In developing countries, the likelihood of women having an abortion is very high, and almost 45% of all globally induced abortions are considered unsafe [5]. Women in Africa are disproportionately affected by the death rate associated with unsafe abortions, despite accounting for 29% of all unsafe abortions, the continent accounts for 62% of all abortion-related deaths [6]. A study conducted in 12 East African countries found that around 5.96% of reproductive-aged women had a history of abortion [7]. An estimated 25 induced abortions per 1,000 women aged 15–44 were performed in Rwanda in 2009 [8].

It is well documented that abortion-related complications can result in severe morbidity if they are not treated promptly, and information and services should be provided accurately to reduce these delays. Thus, to provide safe abortion and contraception services, countries need to implement context-specific programs [9], including CAC which was recently included in the list of essential healthcare services. A wide range of healthcare workers can perform an abortion using medication or surgical procedures effectively [10]. Providers of CAC provide information, manage abortions, and provide post-abortion care. This includes miscarriage care (spontaneous and missed abortions), induced abortion (deliberately interrupting an ongoing pregnancy medically or surgically), incomplete abortion, and fetal death (intrauterine fetal demise) [1]. However, due to the knowledge and skills’ gap observed among healthcare workers including nurses and midwives, pre-service training must be improved to equip them with the necessary skills and knowledge to perform CAC [11,12]. Despite this recommendation to improve pre-service training about CAC some studies have found that the nursing and midwifery educators demonstrate a lack of will to teach CAC [9] and it has been found that this is mostly related to cultural and beliefs factors [13]. This of course lead to inadequate training of students.

In Rwanda, nurses and midwives continue to experience difficulties when providing CAC [14,15]. In order to find a sustainable solution, the Rwandan government, in collaboration with different stakeholders, is focusing on enhancing women’s access to sexual reproductive health services [16–18]. Providing CAC is a new procedure in Rwanda as it has been approved by the Government of Rwanda recently. As part of the national CAC implementation plan for 2022–2024, the Ministry of Health, through the Rwanda Biomedical Centre (RBC), is aligning with the updated WHO guidelines for abortion care [19]. This procedure requires equipping nurses and midwives’ knowledge and skills; however, considering that this still a new intervention being integrated in most nursing and midwifery curriculums, nurses and midwives providing CAC have not learned this in classrooms, some have benefited off job trainings or on job trainings. Yet, no studies have been done in Rwanda to evaluate perceived self-efficacy to teach CAC among teaching faculty from higher learning institutions (HLIs) in Rwanda. Therefore, our study aims to evaluate the perceived self-efficacy to teach CAC among nursing and midwifery faculty in HLIs in Rwanda.

## Materials and methods

### Study design and setting

This study was a mixed-methods design with two phases; quantitative and qualitative [20]. In our explanatory sequential design, we collected quantitative data first, followed by qualitative data, and this allowed us to understand quantitative data further using qualitative data [20]. It was conducted all HLIs (six accepted and one declined) which train nurses and midwives in Rwanda and only one is public [21].

### Study population and sampling strategy

#### Quantitative

This study used total population sampling technique. We invited all 136 nursing and midwifery full-time teaching faculty who actively teach students in classrooms, simulation labs, and clinical instruction to participate in the study. Exclusion criteria were those who are mostly involved in administrative roles and part-time teaching faculty. Among 136 study participants, 14 participated in the pilot phase of the instrument and were not included in the final sample. Thus, 122 study participants were eligible to participate; however, only 98 consented to participate.

#### Qualitative

At the end of each questionnaire there was a question inviting participants to join the qualitative study. All study participants who wished to join the FGDs were invited—however, only 32 consented to participate and were divided into four groups.

### Data collection instruments

#### Quantitative

In quantitative part, the Self-Efficacy Towards Teaching Inventory for Nurse Educators (SETTI-NE) [22] was adapted and piloted on 14 study participants who were not included in the final sample and the reliability Cronbach’s Alpha of 0.951. The instrument comprised of two sections: a socio-demographic section composed of 8 sub-questions and the second section of CAC teaching self-efficacy composed of 54 items evaluating course preparation, instructor behavior and delivery, evaluation and examination, and clinical practice. For each item, the study participants ranked their confidence using a scale arranged from not confident (0), somewhat confident (1), moderately confident (2), and completely confident (3).

#### Qualitative

The research team with the guidance from mentors developed an interview guide to more fully understand self-efficacy in teaching CAC among nursing and midwifery faculty. The following invitation questions were used to facilitate the FGDs:

1. *Can you tell me how you feel when teaching CAC*?
2. *Tell me about the challenges you face when teaching CAC*
3. *What factors influence your teaching ability of CAC in the classroom*, *simulation lab and clinical practice*?
4. *What factors hinder your ability to teach CAC in classrooms*, *simulation labs*, *and clinical practice*?
5. *How do personal beliefs impact your ability to teach CAC*?
6. *What do you think can improve your teaching of CAC*?
7. *What else would you recommend to improve teaching CAC*?

#### Data collection procedures

The data collection procedure was carried from August 02 –September 28, 2022. Six research team members conducted data collection activities. The Pre-Publication Support Service (PREPSS) provided authors with training regarding project management, data collection procedures, and ethical considerations. Two senior mentors in the project mentored the research team members regarding the use of data collection instruments and exercising reflexivity during qualitative data collection. A meeting was held between the research team members to divide the tasks and assign them to individual team members. Data collection was done in person and two data collectors spent five days at each study site.

After getting ethical approval from the University of Rwanda, College of Medicine and Health Sciences Institutional Review Board (UR-CMHS-IRB No 335/CMHSIRB/2022), the research team sought authorization from seven HLIs authorities to access the study participants. However, one institution declined to participate. The study participants were approached and invited to participate in a study after being given deep information on the study. KoboToolbox was used to fill out the questionnaires. Interested participants were invited to join FGDs. A total of four groups of six to eight participants were formed. The FGDs held in private place and were conducted in English. The average length of the interview in qualitative phase was 51mins.

### Ethical considerations

Ethical clearance was obtained from the UR-CMHS-IRB No 335/CMHSIRB/2022 and presented to all HLIs to request permission to conduct the study at their sites.

All participants provided their informed consents after a detailed explanation of the aim and the conduct of the study as well as their role and potential risks. Participants were allowed to withdraw from the study at any time before the analysis phase.

### Analysis

#### Quantitative

Data were retrieved from KoboToolbox and exported to SPSS version 26. Frequencies, percentages, means, and standard deviations were used in the descriptive analysis. The components of self-efficacy were assessed using a scale assessment from not confident (0), somewhat confident (1), moderately confident (2) *and* completely confident (3). The scores were aggregated to calculate an overall average. Then those who scored mean or higher were classified as having adequate self-efficacy and those scored below the mean as in-adequate self-efficacy. We determined the association between sociodemographic characteristics and faculty-perceived self-efficacy using Chi-square test. A p-value of 0.05 was set as the significance level.

#### Qualitative

The interview was transcribed verbatim by three research team members (JB, JBHH, DYNU) who are fluent in Kinyarwanda and English. Dedoose was used to organize data and coding. Thematic analysis was used to identify and report the emerging themes [23]. Three research team members participated in the coding process and held meetings regularly to agree on codes. After generating the initial list of codes, all the research team members held a meeting to agree on the definitions given to codes and generate themes. Two mentors provided regular feedback regarding the sub-themes and themes. A final meeting was organized to confirm the themes and reporting.

## Results

### Quantitative

#### Distribution of socio-demographic characteristics and their associations with perceived self-efficacy in teaching CAC

More than half of the study participants 46(54.1%) were aged 30 to 39 and majority 53(64.2%) were females. Most 80.0% and 72.9% of the respondents were from urban and public institution respectively. About three quarter 64(75.3%) were holders of Master’s degree. Slightly over half 51(60.0%) were nurses and 54(63.5%) were academically belong to assistant lecturers. Considerable percentage (54.1%) worked as nursing educators for more than 10 years whereas 55.3% had less than 6 years of experience in clinical setting. About 56.5% of the respondents also had teaching experience in other fields Table 1. The proportion of respondents with adequate self-efficacy for teaching CAC was 58.8%. Further, the Table 1 shows that gender, education background and working experience in nursing education were associated with perceived self-efficacy of teaching ability for CAC (p < 0.05) where males, midwives and those worked more than 15 years of experience had higher self-efficacy.

**Table 1:** Distribution of sociodemographic characteristics and their association with perceived self-efficacy in teaching CAC.

Attributes	Total, n(%)	Adequate self-efficacy (n = 50)	In-adequate self-efficacy (n = 35)	p value
**Age in years**				
30 to 39	46(54.1)	23(50.0)	23(50.0)	0.073
40 and above	39(45.9)	27(69.2)	12(30.8)	
**Gender**				
Male	32(37.6)	24(75.0)	8(25.0)	0.019
Female	53(62.4)	26(49.1)	27(50.9)	
**Residence/locality**				
Urban	68(80.0)	40(58.8)	28(41.2)	1.000
Rural	17(20.0)	10(58.8)	7(41.2)	
**Type of institution**				
Public institution	62(72.9)	35(56.5)	27(43.5)	0.466
Private institution	23(27.1.0)	15(65.2)	8(34.8)	
**Level of education**				
Bachelor’s degree	21(24.7)	9(42.9)	12(57.1)	0.087
Master’s degree	64(75.3)	41(64.1)	23(35.9)	
**Educational background**				
Midwife	34(40.0)	26(76.5)	8(23.5)	0.007
Nurse	51(60.0)	24(47.1)	27(52.9)	
**Academic Rank**				
Tutorial assistant	27(31.8)	12(44.4)	15(55.6)	0.064
Assistant lecturer	54(63.5)	34(63.0)	20(37.0)	
Lecturer	4(4.7)	4(100.0)	0(0.0)	
**Working experience in nursing education**				
<6 years	10(11.8)	4(40.0)	6(60.0)	0.047
6 to 10 years	29(34.1)	14(48.3)	15(51.7)	
11 to 15 years	32(37.6)	20(62.5)	12(37.5)	
>15 years	14(16.5)	12(85.7)	2(14.3)	
**Working experience in clinical setting**				
<6 years	47(55.3)	27(57.4)	20(42.6)	0.193
6 to 10 years	16(18.8)	7(43.8)	9(56.3)	
>10 years	22(25.9)	16(72.7)	6(27.3)	
**Teaching experience in other fields**				
Yes	48(56.5)	28(58.3)	20(41.7)	0.917
No	37(43.5)	22(59.5)	15(40.5)	

#### Descriptive statistics for the components of self-efficacy in teaching ability

Detailed description using frequency, percentage, mean and standard deviation for each item and component of self-efficacy is presented in S1 Table. The mean and median score of perceived self-efficacy for course preparation and instructor behavior and delivery were higher compared to evaluation/examination and clinical practices Table 2.

**Table 2:** Descriptive statistics on perceived self-efficacy across four domains.

Items	Number of items	Mean score ± Std deviation	Median (IQR)	Max score
Self-efficacy in course preparation	10	20.4±7.6	21(17–26)	30
Self-efficacy in instructor behaviour and delivery	14	29.5±10.3	31(27–37.5)	42
Self-efficacy in evaluation and examination	14	23.6±15.1	28(14–37)	42
Self-efficacy in clinical practice	16	26.7±16.4	32(15.5–42)	48
Overall self-efficacy score	54	100.3±44.1	108(62–137)	162

#### Qualitative

During qualitative analysis, four themes were identified: a) variability in confidence levels to teach CAC; b) readiness about teaching CAC; c) facilitators of teaching CAC; and d) institutional barriers to teach CAC.

#### Variability in confidence levels to teach CAC

Generally, the faculty that participated in this study were either involved in teaching students in classroom, simulation lab or in clinical practice. Few of the participants mentioned being involved in teaching only in classroom but not in clinical. Although, when asked about their insights towards their self-efficacy to teach CAC, the respondents had varied statements.

Seven participants expressed being confident, eight declared being very confident to provide both theory and practice, ten respondents to be only confident in theory but not in practice or in some components of CAC while others reported that they were not confident at all. One participant said: *“I am not confident at all to teach CAC*.*”* N04 Nurse Faculty. Other study participants highlighted that they were very confident to teach CAC as evidenced by the following quotes:

*So for me*, *I am very confident when I teach because I know that safe abortion or CAC can be recommended for anyone who can have an indication for that or just who request that*, *I believe in that I strongly believe in rights about abortion*. M01 Midwife faculty

Another study participant mentioned that since CAC is a new concept, they are confident in teaching some of its components as noted, *“I cannot say that I am confident enough to teach all CAC components*. *I can teach some contents but not all*.*”* N03 Nurse faculty In this similar context another participant added, *“I can teach theory but I do not feel confident in practice*.*”* N04 Nurse faculty

#### Readiness about teaching CAC

When asked about their readiness to teach CAC in classroom, simulation labs and clinical settings, the majority respondents expressed various responses. For instance, one participant reported:

*It is difficult to me to even talk about safe abortion*, *because of the society in which we are living in*. *If I emphasize on that*, *maybe it will be an advertisement…*… *So personally*, *a part of knowledge that I am lacking*, *I do not have a will on top of that*. N05 Nurse Faculty

When asked why teaching faculty are not ready to teach CAC, the mentioned religious beliefs as the main barrier as evidenced by the following quotes:

*I am a pastor’s wife and sings is choir*. *Those are the barriers that can prevent me to teach CAC*. *Those are my personal believes*, *being mama pastor*, *singing in choir and being a protestant*. *With that I cannot teach that*. *I can’t kill*, *I have to preach how to save but not how to kill*. N02 Nurse Faculty

*Teaching about CAC*, *I somehow don’t know where I belong now*. *I received the training on safe abortion but according to my beliefs*, *I was not even understanding why they are giving this course…I will not teach those kind of theories*. *For practices*, *I cannot teach sincerely those practices*.*”* N08 Nurse Faculty

In this similar vein, other participants added that they can perform or teach CAC when it is putting a woman in danger as illustrated in the following quotes:

*Personally*, *for post abortion care either from induced or spontaneous abortion*, *I do not have any judgment*. *But for an induced abortion*, *I cannot do it*, *unless if there is a life threatening situation or a malformed baby*, *otherwise I will be feeling like committing a sin if I induce someone’s abortion unless if there are those specific conditions*. N17 Nurse Faculty

*Personally I do not have any judgement regarding CAC if it is a life threatening condition*. *But when it comes to induced one*, *I am not comfortable in teaching nor doing it*. *If I am allocated I will teach it but*, *I am not comfortable*. M05 Midwife faculty

#### Facilitators of teaching CAC

The reasons of teaching CAC that were underlined by respondents mainly were received training, well equipped skills laboratory and exposure in health settings. One study participant said: “*We got training and we were facilitated with CIRHT*. *They are [some] equipment that have been supplied and we got training about all those concepts*.*”* N09 Nurse Faculty In this similar vein, another participant added: *“I have enough training about post abortion care*, *and also am a lecturer…*. *I have this like unit of abortion because it is included in complication of early pregnancy*.*”* N13 Nurse faculty Even though they were trained, there are a few participants who reported that they did not receive any kind of CAC training or had not been updated or not had not been clinically exposed as evidenced by the following quotes:

*As my colleagues have said that there is lack of trainings about CAC*, *me too I am not trained about it*. *But if I am trained this could be good because it saves lives … So what is needed here*, *we need training in order*, *so what can hinder me for teaching is that I do not have training on it*. M06 Midwife faculty

*One challenge is the lack of CAC training*. *This influences negatively my teaching ability*. *I am not updated*, *I am not exposed*, *so I don’t know what to provide to students*. N07 Nurse faculty.

During the discussion, it was not that some institutions do not receive CAC trainings due to lack of partners with their HLIs. Those from HLIs which work with partners reported being confident and acknowledged the role of this partnership.

*Institutional barriers to teach CAC*. The study participants reported that faith based health settings where students are sent for clinical practices as the main challenge as explained, *“Sometimes students are allocated to faith based settings and do not get the opportunity to practice CAC”*. M12 Midwife faculty This was a challenge since there are HLIs which have a memorandum of understanding with those faith based institutions to send students. Lack of CAC content in nursing and midwifery curriculum affect how nursing and midwifery faculty teach. For instance, one study participant said, *“…the curriculum should be revised first to accommodate this concept [CAC]*. *Currently*, *it [CAC] does not appear anywhere*.*”* N18 Nurse Faculty A few of participants have mentioned lack of the appropriate materials in simulation lab to use when teaching CAC as explained by one participant: *“Sometimes you may want to teach students and you do not find the CAC materials*. *It’s better the skills lab are well equipped*.*”* N07 Nurse Faculty

## Discussion

The purpose of this study, which used a mixed method design, was to evaluate perceived self-efficacy and readiness to teach CAC among nursing and midwifery faculty in Rwanda. The quantitative approach demonstrated that more than half 58.8% had adequate self-efficacy in teaching CAC. Factors affecting self-efficacy to teach CAC were gender (being male), years of experience in nursing education, and professional status as a midwife. Besides, the qualitative study identified factors that support effective teaching of CAC in clinical and classroom settings, including exposure to health settings, obtaining training, having a well-equipped skills lab, and having high confidence. On the other hand, the qualitative study highlighted certain barriers to teaching CAC, including unfavorable personal and religious convictions, a lack of CAC content in nursing and midwifery curricula, and a dearth of simulation laboratory resources.

Our findings revealed that of all the 85 respondents, only 58.8% had adequate self-efficacy in teaching CAC. This indicates that some teachers lack confidence in their ability to teach CAC effectively. This lack of self-efficacy could have a significant impact on student learning outcomes. Schools should ensure teachers have the necessary training and resources to feel confident when teaching CAC. These concerns have also been raised in another study conducted in Ghana, where the midwifery tutors had a low level of knowledge regarding abortion care [13].

In the present study, males are more likely to have more perceived self-efficacy for teaching CAC compared to their female counterparts. These findings are similar to other studies conducted in other settings [24,25]. There is evidence that male healthcare professionals are more likely than females to have favorable attitudes toward safe abortion care [12]. This may explain why Rwandan males are more prepared and have higher self-efficacy in teaching CAC. Some studies found that gender does not affect one’s self-efficacy [26,27] while others found the relationship between gender and perceived self-efficacy [28,29]. It appears that gender can play a role in how a person perceives their self-efficacy. However, further research is needed to better understand the exact relationship between gender and self-efficacy in teaching CAC.

The study also discovered that compared to nursing professionals, midwifery professionals had considerably higher perceived self-efficacy. Evidence also shows that midwives displayed increased skill and confidence in CAC practice and instruction compared to other professions [30]. Similar to this, a study carried out in Ethiopia revealed that midwives were more knowledgeable about safe abortion care than nurses [12]. This might be because midwives work with pregnant women on a regular basis, which helps them gain a better knowledge of abortion-related issues. Nursing faculty should receive extensive pre-and in-service CAC training, and their curriculums should reflect these differences. In addition, nursing faculty should also be provided with additional guidance and support to ensure they have the necessary knowledge and skills to teach CAC.

The reported self-efficacy was significantly higher among respondents with more than 15 years of experience. The social learning theory posits that one’s self-efficacy can change as the career progresses [31]. A growing body of knowledge found that nurse educators develop self-efficacy by time and teaching experience [32,33]. The FGD participants also reported that exposure to health environments and training help to effectively teach CAC. These findings are consistent with other studies which revealed the role of continuous professional development on teaching self-efficacy [32,34,35]. This training and exposure hand-on as well as the longer years of experience might have played a role for the high level of perceived teaching self-efficacy for clinical practice among nurses and midwives.

Despite the aforementioned facilitators, this study also found that negative religious and personal beliefs towards abortion were the common barriers to teaching effective CAC similar to the study carried out in Ghana [13]. Other studies revealed that healthcare professionals struggled to deliver safe abortion care due to their moral and religious beliefs [36,37]. To address these barriers, values clarification and attitudinal transformation (VCAT) training have been proven to be effective for abortion providers to examine their values and attitudes toward abortion and the consequences when their patients cannot access abortions [38,39]. Therefore, this study’s findings highlight the importance of similar training for faculty teaching CAC to ensure they are equipped with the knowledge and skills needed to provide the highest possible support to their students. For this training to succeed, nursing and midwifery faculty should be encouraged to reflect on their own assumptions and beliefs to ensure that teaching CAC is unbiased and comprehensive. Furthermore, faculty should have access to resources and support to cope with potential moral and ethical dilemmas that may arise.

Lack of CAC content in nursing and midwifery curriculum was also a reported barrier of self-efficacy in teaching CAC, consistent with another Ghana study [13]. It’s understandable that incorporating CAC content into the curriculum can increase faculty self-efficacy in teaching CAC, leading to better student learning. Lastly, the respondents reported that lack of simulation laboratory materials hinder their self-efficacy in teaching CAC. In obstetrics, simulation training has been found to be effective in previous studies [40,41]. Simulator training can be used as a tool for learning fundamental skills, can help with counseling and communication, and can encourage discussion [42]. It is well documented that well-equipped simulation laboratories contribute to effective teaching [43,44]. Therefore, equipping simulation laboratories should be a top priority for HLIs in Rwanda to ensure faculty have access to the resources they need to provide quality education. This will also provide students with the opportunity to practice their CAC skills in a simulated environment before conducting real medical procedures.

### Strengths and limitations

This study has many strengths. First, one of the strengths is the use of mixed methods to explore teaching of CAC by cross-validating and triangulating quantitative and qualitative data. By using this design, we were able to create a more complete picture of how self-efficacy was perceived and described in teaching CAC, thereby enabling a better and deeper understanding of this central concept. Second, we considered the total population sampling from all HLIs in Rwanda either private and public or urban and rural which is satisfactory for findings generalization. However, this study has limitations. One institution and some faculty refused to participate, contributing to the non-response bias [45]. The non-response bias may have affected the study’s overall results; as non-respondents may have different attitudes or opinions regarding teaching CAC than respondents. Lastly, another limitation might be social desirability, which could induce study participants to over or underreport their CAC teaching experience [46]. Therefore, it is important to consider non-response and social desirability biases when interpreting the findings from this study. Future research should include nursing leadership, assess the capacity of curriculums and skills laboratories, and maximize the participation to gain a comprehensive understanding of self-efficacy about teaching CAC in HLIs.

## Conclusion

The findings of this study revealed that 41.2% of faculty had inadequate self-efficacy in teaching CAC. Being a nurse, being female and having a few years of nursing education experience were significantly associated with low self-efficacy in teaching CAC. The findings highlighted specific barriers to teaching CAC, including unfavourable personal and religious beliefs, a lack of CAC content in nursing and midwifery curricula, and a dearth of simulation laboratory resources. Therefore, to ensure that faculty are confident in teaching CAC, there is an urgent need for HLIs and other stakeholders to focus on factors supporting effective CAC teaching in clinical and classroom settings, including exposure to CAC practices in health settings, providing VCAT training, updating the curricula content, and having well-equipped simulation laboratories. Moreover, emphasis should be put on nurses and those with a few years of working experience in nursing education. Faculty should be provided with mentorship and professional development opportunities.

## Supporting information

S1 Table Distribution and descriptive statistics among the items of CAC teaching self-efficacy. (DOCX)
