# Supplementary material for: Perceived self-efficacy to teach comprehensive abortion care among nursing and midwifery faculty in higher learning institutions in Rwanda: A mixed method study
Source: PLoS One. 2024 Mar 18;19(3):e0300542. doi: 10.1371/journal.pone.0300542 (PMC10947688; doi:10.1371/journal.pone.0300542)
Supplement: S1 Table — (DOCX) [file pone.0300542.s001.docx]

**Appendix 1: Distribution and descriptive statistics among the items of CAC teaching self-efficacy**

| **Statements** | **NC, n(%)** | **SC, n(%)** | **MC, n(%)** | **CC, n(%)** | **Mean** | **Std Dev** |
| --- | --- | --- | --- | --- | --- | --- |
| **Self-efficacy and ability in course preparation** |  |  |  |  |  |  |
| State goals and objectives clearly | 6(7.1) | 14(16.5) | 42(49.4) | 23(27.1) | 1.96 | 0.85 |
| Plan teaching methodologies | 4(4.7) | 19(22.4) | 32(37.6) | 30(35.3) | 2.04 | 0.88 |
| Write a course syllabus | 8(9.4) | 14(16.5) | 36(42.4) | 27(31.8) | 1.96 | 0.93 |
| Plan discussions (in class or online) | 6(7.1) | 12(14.1) | 39(45.9) | 28(32.9) | 2.05 | 0.87 |
| Plan teaching and learning activities | 6(7.1) | 10(11.8) | 40(47.1) | 29(34.1) | 2.08 | 0.86 |
| Select resources to support student learning | 5(5.9) | 12(14.1) | 41(48.2) | 27(31.8) | 2.06 | 0.84 |
| Select relevant readings | 6(7.1) | 10(11.8) | 38(44.7) | 31(36.5) | 2.11 | 0.87 |
| Develop student assignments | 6(7.1) | 11(12.9) | 39(45.9) | 29(34.1) | 2.07 | 0.87 |
| State grading criteria | 6(7.1) | 8(9.4) | 43(50.6) | 28(32.9) | 2.09 | 0.84 |
| Develop teaching strategies that promote critical thinking | 8(9.4) | 8(9.4) | 43(50.6) | 26(30.6) | 2.02 | 0.89 |
| **Self-efficacy in instructor behavior and delivery** |  |  |  |  |  |  |
| Deliver teaching methodologies | 7(8.2) | 12(14.1) | 39(45.9) | 27(31.8) | 2.01 | 0.89 |
| Select and use a variety of teaching strategies | 7(8.2) | 13(15.3) | 41(48.2) | 24(28.2) | 1.96 | 0.88 |
| Initiate discussion with students (in class or online) | 7(8.2) | 9(10.6) | 38(44.7) | 31(36.5) | 2.09 | 0.90 |
| Draw students into discussions (in class or online) | 5(5.9) | 11(12.9) | 44(51.8) | 25(29.4) | 2.05 | 0.82 |
| Communicate at a level that matches student's ability to comprehend | 6(7.1) | 7(8.2) | 51(60.0) | 21(24.7) | 2.02 | 0.79 |
| Ask open-ended, stimulating questions | 5(5.9) | 11(12.9) | 39(45.9) | 30(35.3) | 2.11 | 0.85 |
| Recognize and respect individual differences | 6(7.1) | 8(9.4) | 37(43.5) | 34(40.0) | 2.16 | 0.87 |
| Manage student disagreements with instructor | 7(8.2) | 9(10.6) | 38(44.7) | 31(36.5) | 2.09 | 0.90 |
| Communicate consistently both verbally and non-verbally | 6(7.1) | 7(8.2) | 37(43.5) | 35(41.2) | 2.19 | 0.87 |
| Show respect for student ideas and abilities | 6(7.1) | 2(2.4) | 34(40.0) | 43(50.6) | 2.34 | 0.84 |
| Respond appropriately to students’ questions | 8(9.4) | 7(8.2) | 35(41.2) | 35(41.2) | 2.14 | 0.93 |
| Respond to student emotional reactions in class | 6(7.1) | 5(5.9) | 44(51.8) | 30(35.3) | 2.15 | 0.82 |
| Integrate readings and teaching methodologies | 6(7.1) | 11(12.9) | 46(54.1) | 22(25.9) | 1.99 | 0.82 |
| Initiate discussion with a student with a failing grade | 5(5.9) | 11(12.9) | 36(42.4) | 33(38.8) | 2.14 | 0.86 |
| **Self-efficacy in evaluation and examination** |  |  |  |  |  |  |
| Construct exam questions that require integration of content, critical thinking and self-expression | 21(24.7) | 13(15.3) | 34(40.0) | 17(20.0) | 1.55 | 1.08 |
| Construct test questions that are at cognitive domain of apply or higher (apply, analyze, evaluate, create) | 19(22.4) | 15(17.6) | 33(38.8) | 18(21.2) | 1.59 | 1.06 |
| Develop a test plan | 19(22.4) | 15(17.6) | 34(40.0) | 17(20.0) | 1.58 | 1.05 |
| Score exams and interpret results | 20(23.5) | 13(15.3) | 25(29.4) | 27(31.8) | 1.69 | 1.16 |
| Evaluate student assignments | 19(22.4) | 12(14.1) | 25(29.4) | 29(34.1) | 1.75 | 1.15 |
| Utilize exams as learning tools | 19(22.4) | 16(18.8) | 23(27.1) | 27(31.8) | 1.68 | 1.15 |
| Provide constructive feedback on exams and assignments | 18(21.2) | 15(17.6) | 20(23.5) | 32(37.6) | 1.78 | 1.17 |
| Identify a student having academic/clinical practice difficulty | 18(21.2) | 15(17.6) | 26(30.6) | 26(30.6) | 1.71 | 1.12 |
| Direct or advise students who are experiencing academic/clinical practice difficulty | 18(21.2) | 15(17.6) | 28(32.9) | 24(28.2) | 1.68 | 1.10 |
| Conclude a student's clinical practice performance is failing | 18(21.2) | 16(18.8) | 28(32.9) | 23(27.1) | 1.66 | 1.10 |
| Utilize self-evaluation to improve teaching | 18(21.2) | 16(18.8) | 20(23.5) | 31(36.5) | 1.75 | 1.16 |
| Arrange for constructive feedback and suggestions from peers | 18(21.2) | 14(16.5) | 26(30.6) | 27(31.8) | 1.73 | 1.13 |
| Use feedback from students to improve teaching | 17(20.0) | 14(16.5) | 24(28.2) | 30(35.3) | 1.79 | 1.14 |
| Evaluate the expected outcomes of a course | 20(23.5) | 12(14.1) | 25(29.1) | 28(32.9) | 1.72 | 1.16 |
| **Self-efficacy in clinical practice** |  |  |  |  |  |  |
| Set clinical practice expectations that are appropriate for the level of the learner in patient care areas | 21(24.7) | 15(17.6) | 24(28.2) | 25(29.4) | 1.62 | 1.15 |
| Modify clinical teaching strategies based on learner's level of performance | 20(23.5) | 14(16.5) | 29(34.1) | 22(25.9) | 1.62 | 1.11 |
| Ask questions in a clinical practice setting that stimulate problem-solving | 19(22.4) | 15(17.6) | 23(27.1) | 28(32.9) | 1.71 | 1.15 |
| Provide constructive feedback in a supportive manner regarding clinical practice performance | 19(22.4) | 13(15.3) | 26(30.6) | 27(31.8) | 1.72 | 1.14 |
| Demonstrate confidence in the student | 19(22.4) | 12(14.1) | 22(25.9) | 32(37.6) | 1.79 | 1.18 |
| Assist student in new patient care situations | 20(23.5) | 14(16.5) | 26(30.6) | 25(29.4) | 1.66 | 1.14 |
| Stimulate the student’s interest to learn professional behavior and competence | 19(22.4) | 15(17.6) | 24(28.2) | 27(31.8) | 1.69 | 1.15 |
| Adjust clinical practice assignments to individual's level of performance and confidence | 19(22.4) | 20(23.5) | 27(31.8) | 19(22.4) | 1.54 | 1.08 |
| Use evaluation criteria to appraise student's clinical practice performance | 19(22.4) | 17(20.0) | 28(32.9) | 21(24.7) | 1.60 | 1.09 |
| Record and use anecdotal observations as part of clinical practice evaluation | 22(25.9) | 16(18.8) | 28(32.9) | 19(22.4) | 1.52 | 1.11 |
| Integrate best practices into simulation-based experience | 18(21.2) | 15(17.6) | 28(32.9) | 24(28.2) | 1.68 | 1.10 |
| Develop expected outcomes for simulation-based experiences | 17(20.0) | 18(21.2) | 28(32.9) | 22(25.9) | 1.65 | 1.08 |
| Modify simulation facilitation to student’s level of experience and competence | 18(21.2) | 16(18.8) | 33(38.8) | 18(21.2) | 1.60 | 1.05 |
| Use debriefing after a simulation-based experience to encourage learning | | 19(22.4) | 45(52.9) | 21(24.7) | 2.02 | 0.69 |
| Use simulation expected outcomes as basis for student evaluation | 18(21.2) | 17(20.0) | 27(31.8) | 23(27.1) | 1.65 | 1.10 |
| Provide a supportive learning environment for the simulation-based experience | 19(22.4) | 16(18.8) | 25(29.4) | 25(29.4) | 1.66 | 1.13 |
| **NC = Not confident; SC = Somewhat confident’, MC = Moderately confident, CC = Completely confident** | | | | | | |
